# Advances and Challenges in the Battery Thermal Management Systems of Electric Vehicles

**DOI:** 10.3390/ma18204718

**Published:** 2025-10-15

**Authors:** Tianze Wen, Zhequan Zhou, Yongyi Zhang, Xiaomei Xu

**Affiliations:** College of Automobile and Traffic Engineering, Nanjing Forestry University, Nanjing 210037, China; 18361217529@163.com (T.W.);

**Keywords:** lithium-ion battery, battery thermal management systems, phase change materials, liquid cooling, heat pipe, current challenges

## Abstract

Battery Thermal Management Systems (BTMS) are essential for ensuring the performance, safety, and longevity of lithium-ion batteries (Li-ion) in electric vehicles (EVs). First, this review examines the current state of BTMS technologies, focusing on three thermal management strategies: passive, active, and hybrid thermal management strategies. Passive thermal management strategies, such as using phase change materials (PCM) or heat-conductive materials, offer simplicity and low energy consumption but are limited in high-power applications. The active thermal management strategies, including forced air cooling and liquid cooling, provide superior heat dissipation but require complex design and higher energy input. The hybrid thermal management strategies combine the advantages of passive and active strategies, providing a more suitable solution for the thermal management of lithium-ion batteries under diverse operating conditions. Second, the review also highlights challenges posed by high-energy density batteries, fast charging, and emerging battery technologies like solid-state and lithium–sulfur batteries. Finally, the technical summary draws from the research status of BTMS and future development directions are proposed.

## 1. Introduction

Lithium-ion batteries (Li-ion), recognized as the prevailing energy storage solution for electric vehicles (EVs), serve as a critical component of the EV power system. However, their performance, safety, and longevity are profoundly influenced by operating temperature. Effective thermal management not only governs charge/discharge efficiency but also dictates battery safety and durability. Therefore, battery thermal management systems (BTMS) have become a crucial component of EVs. A large number of researchers have conducted in-depth research on BTMS.

During charge and discharge cycles, Li-ion batteries inevitably generate substantial amounts of heat due to internal electrochemical reactions and ohmic resistance. The accumulated thermal energy leads to considerable temperature rises within battery cells [1,2,3]. A temperature that is too high will cause the battery to face overheating risks. Here are several common overheating risks: (1) thermal runaway, which is one of the most serious consequences when a battery overheats. When the battery temperature reaches a critical threshold, the chemical reactions inside the battery will intensify, releasing more heat, leading to a further temperature rise and forming a self-accelerating thermal reaction process [4]. Thermal runaway may cause battery fires or even explosions, posing a serious threat to vehicle and personnel safety. (2) Performance degradation: Elevated temperatures hasten battery deterioration, particularly causing marked declines in capacity and charge/discharge efficiency [5]. Excessive heat promotes electrolyte decomposition and gas formation, potentially causing battery swelling, leakage, or catastrophic internal short-circuits, all of which severely degrade performance and safety. (3) Fire risk: Overheating of the electric vehicle battery can not only cause damage to the battery itself but may also lead to a fire. When the battery temperature exceeds the safe range, thermal runaway may occur inside the battery, ultimately leading to the occurrence of a fire [6]. Battery fires not only cause property damage but may also pose a threat to the safety of drivers’ lives. Under demanding operational regimes such as accelerated charging and high-load discharging, the substantial heat generated within batteries often exceeds the dissipation capacity, leading to a rapid temperature rise. Inadequate thermal management of such temperature escalation may potentially trigger thermal runaway.

**Figure 1:**
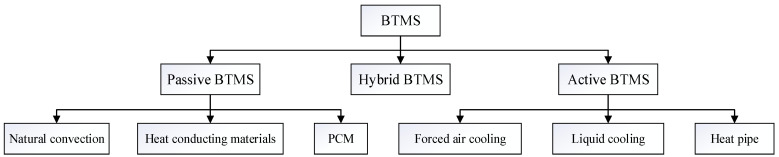
Classification of BTMS.

BTMS are engineered to regulate pack temperatures within the ideal operational range, ensuring optimal electrochemical performance and longevity [7,8,9]. BTMS comprise three core subsystems: thermal control algorithms, heat transfer media, and temperature monitoring networks. According to different methods of implementing thermal management, BTMS can be divided into two basic types, namely active and passive BTMS. Passive BTMS rely on natural convection and conductive heat-transfer principles, dissipating thermal energy to the ambient environment through optimized battery pack geometry and thermal interface materials. Though this approach minimizes system complexity and power consumption, its limited heat dissipation capacity proves inadequate for high-power applications with stringent thermal requirements. Active BTMS employ forced convection mechanisms to extract battery-generated heat actively. Due to the difficulty of achieving ideal cooling effects with a single thermal management method, hybrid BTMS, combining active and passive thermal management methods, have been proposed. The classification of BTMS is shown in Figure 1. In the figure, PCM refers to the phase change materials.

The design of BTMS affects three key performance indicators of vehicles, namely driving range, charging efficiency, and power output characteristics. First of all, the battery operating temperature has a significant correlation with capacity retention and energy conversion efficiency. Compared with ordinary BTMS, optimized BTMS can maximize energy efficiency and expand driving range by up to 25% [4]. Secondly, during charging cycles, the electrochemical heat generation necessitates robust thermal management. Inadequate BTMS designs induce thermal throttling, reducing charge acceptance rates [6]. The last indicator is power output; battery temperature critically influences discharge characteristics, particularly under high-current loads. An optimized BTMS ensures thermal stability, preventing power derating due to excessive heat accumulation and thereby sustaining a rated vehicle performance [10,11].

Additionally, BTMS requirements vary according to battery chemistry. The two predominant battery types in electric vehicles are lithium iron phosphate (LFP) and ternary lithium (NCM) batteries. LFP batteries are extensively utilized in EVs, owing to their superior thermal stability and enhanced safety characteristics. Compared to NCM batteries, LFP batteries impose less stringent thermal management demands, due to their improved high-temperature tolerance and diminished thermal runaway propensity. However, their comparatively lower energy density necessitates optimized thermal regulation to maximize performance and cycle life [12]. In contrast, NCM batteries exhibit a superior energy density, making them ideal for high-performance electric vehicle applications. However, this enhanced energy density introduces more demanding thermal management challenges. NCM batteries demonstrate significant heat generation during fast charging and high-power discharge cycles, necessitating advanced cooling solutions to maintain optimal operating temperatures [13].

This study provides a comprehensive review of the existing technologies for BTMS, analyzes their respective advantages and limitations, and discusses their potential future development directions.

## 2. Passive BTMS

The passive BTMS regulates the temperature of the battery through natural physical processes, such as natural convection, thermal storage of heat-conducting materials, and phase change materials. The main advantages of these technologies are low energy consumption, low cost, and simplicity, but they may face some limitations in the application of high-energy density and fast charging.

Natural convection represents a passive thermal regulation mechanism that leverages buoyancy-driven fluid motion, offering both simplicity in implementation and cost-effectiveness in operation. It uses the natural flow of air to take away the heat generated by the battery. By optimizing the internal arrangement of the battery pack and the design of the battery shell, the air can flow naturally, so as to achieve the effect of heat dissipation. The advantage of natural convection is that it does not need additional energy consumption and does not rely on external energy. The intrinsic limitations of air, namely its low thermal conductivity and inadequate heat dissipation efficiency, generally restrict its use to low-power battery packs or scenarios with minimal thermal loads [14]. When discharging at a low current, a small amount of heat generated by the battery is mainly stored in the material as sensitive heat, which is easy to be dissipated into the environment and dissipated by the natural convection of air; during high-current discharge cycles, the predominant heat generation mechanism shifts to latent heat storage within battery materials. While natural convection suffices for basic cooling needs in early-stage thermal management systems, its inherent inefficiency at low ambient temperatures creates critical limitations: (1) a progressive depletion of thermal storage capacity due to incomplete inter-cycle heat dissipation, and (2) a cumulative temperature rise establishing elevated baseline conditions for subsequent discharges. However, the most basic method of heat dissipation in the early stage of the development of BTMS technology is through natural convection [15].

Heat conducting materials (such as aluminum, copper and graphite) are another common passive cooling technology. They transfer the heat generated by the battery to the radiator or the surrounding environment through good thermal conductivity. The principal advantage of thermal interface materials resides in their ability to facilitate superior heat dissipation, particularly when integrated with a rationally designed battery pack thermal architecture. By accurately designing the thermal conduction path of the battery cell, the heat conduction material can evenly disperse heat [16]. However, the application of heat conducting materials has certain limitations. Because the heat dissipation process is limited by the materials’ own thermal conductivity, some materials with low thermal conductivity have a poor effect in high-power battery packs, and cannot dissipate heat quickly and effectively [17].

**Figure 2:**
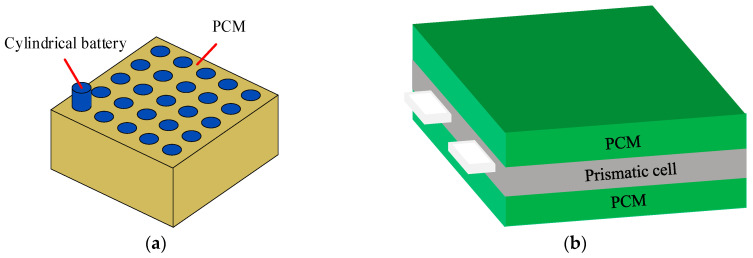
Common structures of PCM. (**a**) PCM structure for cylindrical batteries; (**b**) PCM structure for prismatic batteries.

Phase change materials (PCM) are widely used in building energy conservation, waste heat recovery [18], photothermal conversion and storage [19], battery thermal management [20,21], and other fields because they maintain a constant temperature during the phase change process. When the temperature of the battery module rises, the phase change material absorbs heat in the form of sensible heat, and its temperature rises. Then, the PCM subsequently commences melting, persistently absorbing heat in the form of latent heat, thereby dissipating a considerable amount of thermal energy from the module [22]. For the application of PCM in battery cooling systems, different types of batteries typically use PCM with different structures. For cylindrical batteries, PCM are usually used to wrap them; for square batteries, PCM are generally placed between the battery cells, as shown in Figure 2. As a PCM, paraffin has the advantages of high latent heat and low cost, but the thermal conductivity of pure paraffin is low, so the research mainly focuses on composite PCM.

For the implementation of PCM in BTMS, Ling et al. [23] proposed a thermal management system for inorganic phase change material batteries and a multi-scale encapsulation method, which uses the micro-scale encapsulation of expanded graphite to increase the thermal conductivity of PCM to 4.96 W/m·K. A comparative analysis was conducted between inorganic and organic phase change materials for battery pack thermal management. The results showed that the inorganic PCM were safer and could provide a lower temperature and more uniform thermal environment for the battery pack. Ping et al. [24] proposed a new PCM and fin structure for BTMS with a Li-ion battery module. Numerical simulations were employed to systematically investigate the influence of PCM type, fin thickness, and fin spacing on the thermal performance of battery packs. Weng et al. [25] investigated how various fin designs and fin density arrangements influence phase change material thermal conductivity enhancement, benchmarking the performance against conventional PCM systems without fins. Jiang et al. [26] engineered a composite PCM by incorporating expanded graphite into paraffin wax, systematically evaluating its thermal enhancement effects across varying filler concentrations (5–25 wt%). Their results demonstrated a direct correlation between graphite content and thermal performance, with optimal heat dissipation achieved at 9–20% mass loading. Nasehi et al. [27] explored the use of three-layer PCM around the battery to improve the thermal management performance. The research showed that under adiabatic conditions, the use of single-layer PCM for heat dissipation was better than the use of three-layer PCM. On the contrary, in the presence of air convection, the heat dissipation effect of three-layer PCM was better. In summary, these studies show that the fillers reduce the temperature of the battery packs but increase weight, and multilayer PCM only works well under forced convection. It is worth noting that PCM has not been substantially used in EVs because of weight and safety concerns.

## 3. Active BTMS

This section mainly introduces the related technologies of active BTMS, and reviews the definition, development, advantages, and disadvantages of various technologies, as well as the comparison between different technologies. The active BTMS uses external energy sources (such as fans, pumps, and coolants) to actively regulate the battery temperature. Compared with the passive system, the active system can provide a stronger cooling capacity, which is especially suitable for a high-power battery pack and fast-charging environment.

### 3.1. Forced Air Cooling

**Figure 3:**
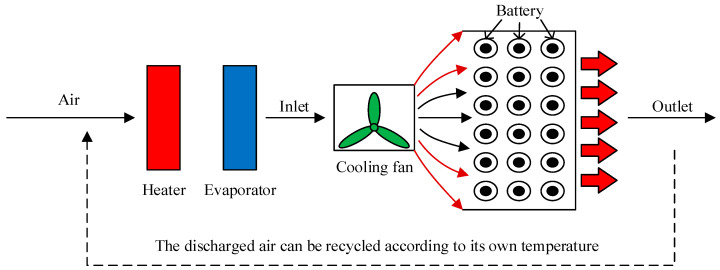
Forced air cooling system.

On the basis of natural air cooling, forced air cooling adds a fan or blower to force the air flow to improve the convective heat transfer efficiency between the battery surface and the air, as shown in Figure 3. This method has a high cooling efficiency and can flexibly adjust the heat dissipation intensity as required, but the system has high energy consumption and noise, and the flow channel needs to be reasonably designed to prevent uneven air distribution from affecting the cooling effect [28,29].

**Figure 4:**
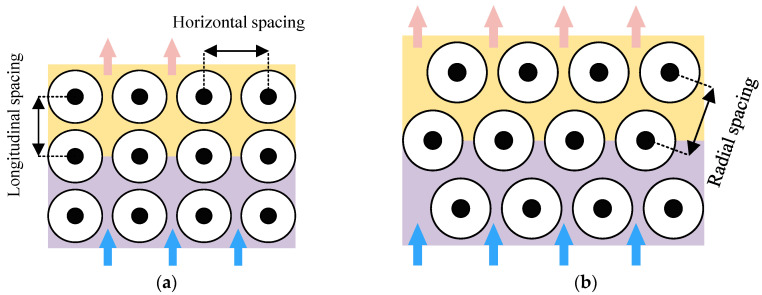
Common arrangement of battery cells under air cooling conditions. (**a**) Aligned arrangement; (**b**) staggered arrangement.

In terms of battery arrangement, Yang et al. [30] compared and analyzed the thermal performance of lithium iron phosphate cylindrical batteries with different arrangement methods, and proposed two methods: aligned arrangement and staggered arrangement, as shown in Figure 4. They also analyzed the influence of longitudinal and transverse spacing on the temperature performance of the two arrangement methods. The study found that under a certain cooling air flow rate, the maximum temperature rise in staggered arrangement batteries is proportional to the longitudinal spacing, while the maximum temperature rise in aligned arrangement batteries is inversely proportional to the longitudinal spacing. Regardless of the arrangement method, an increase in transverse spacing will lead to an increase in battery temperature. Du et al. [31] discussed the air cooling and heat dissipation effects of battery packs under four different battery arrangements, including a square arrangement, a staggered arrangement, and two trapezoidal arrangements. The study found that the best air-cooled effect was achieved when the square arrangement was used and the air inlet was placed at the top of the battery pack. Peng et al. [32] conducted a comparative study on cylindrical battery pack air cooling systems, evaluating different battery arrangement configurations, along with various inlet/outlet positions and quantities in the cooling system, and the results indicate that a layout with a smaller aspect ratio is more beneficial for enhancing cooling performance, while a rational arrangement of the inlet/outlet in the cooling system can effectively mitigate battery heating. Similarly, Kang et al. [33] conducted research on a module composed of 16 battery cells, arranging them into “4 × 4” squares and “2 × 8” rectangles. Research results show that the internal temperature distribution is directly affected by the arrangement of the batteries.

**Figure 5:**
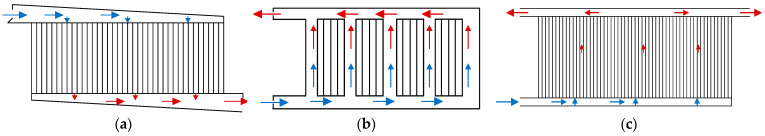
Three different forms of air flow channels. (**a**) Z-shaped; (**b**) U-shaped; (**c**) J-shaped.

In terms of air flow channel design, Sun et al. [34] designed a “Z-shaped” air flow channel with a conical inlet and outlet, and used the optimal Latin hypercube optimization method to optimize its parameters. The experimental data validate the efficacy of the optimized channel geometry in stabilizing the air flow, ensuring superior thermal homogeneity across the battery cells, and achieving a marked reduction in total system pressure loss. Chen et al. [35] also optimized the design of the “Z-type” cooling channel. Newton’s method was used to simulate different air inlet angles and air inlet and outlet widths, and the optimal combination was found through a nested cycle program. The study revealed that adjusting the air inlet angle had negligible impact on enhancing the battery’s thermal dissipation performance, but optimal air channel geometry, particularly precise inlet/outlet width configuration, significantly improves battery temperature uniformity. To enhance cooling efficiency, Lu et al. [36] developed a “U-shaped” cooling channel configuration featuring the top-mounted air inlets and outlets. The experimental results demonstrated that compared to the conventional “Z-shaped” channel layout, this design achieved a 3 K reduction in the maximum temperature difference across the battery. Furthermore, Liu et al. [37] ingeniously combined the “U-shaped” and “Z-shaped” designs and proposed an innovative “J-shaped” cooling channel. They conducted simulation studies on battery modules using three types of channels and found that the “J-shaped” cooling channel can reduce the battery temperature rise by 31.1%. The schematic diagrams of three forms of air flow channels are shown in Figure 5.

**Figure 6:**
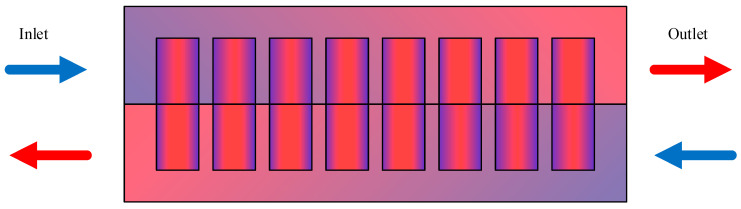
Reverse stratified air flow.

Air flow can be divided into Unidirectional Air Flow (UDAF) and Reverse Layered Air Flow (RLAF) according to the flow path, as shown in Figure 6. Na et al. [38] conducted research on two air flow configuration methods for cylindrical battery modules, and the results showed that compared with UDAF, the RLAF method significantly reduced the temperature difference between batteries and increased the temperature uniformity of battery cells in the battery module. Similarly, R. Mahamud et al. [39] used a reciprocating air flow path in a study to improve the temperature uniformity of lithium-ion batteries for electric vehicles. A numerical simulation showed that compared to unidirectional flow, the reciprocating air flow reduced the battery temperature difference and maximum temperature by approximately 4 °C and 1.5 °C, respectively. In addition, scholars have conducted a structural optimization design on factors such as the shape and position of the flow guide plate inside the battery pack, the number and layout of ventilation openings, and the position of the fan, and designed reasonable control strategies to optimize the cooling effect. E et al. [40] studied the effect of the air flow inlet and outlet on cooling performance, and found that setting ventilation holes on the side of the battery box can achieve a better cooling effect. Battery systems with air-cooled ventilation holes on different sides have a better cooling performance than systems with ventilation holes on only one side. Yu et al. [41] studied the influence of two cooling ducts with independent air intakes and fans on the cooling effect of batteries. One fan was used for cooling, and the other fan was used to improve the air circulation inside the battery. Ultimately, the highest temperature of the battery was maintained at 33.1 °C, greatly reducing the accumulation of heat between individual cells in the battery pack. Chen et al. [42] developed a control strategy based on cell temperature difference, for parallel air-cooled systems to effectively cool battery packs under different operating conditions. The system adopting this strategy can switch flow types in real time when the temperature difference reaches the preset value, reducing the average temperature difference by more than 67%.

In summary, research on forced air cooling mainly involves three aspects, namely battery arrangement, air flow channel design, and air flow path design. At present, the control scheme for forced air cooling tends to use “J-shaped” cooling channels and RLAF methods, and the specific arrangement of the batteries should be determined according to the spatial structure of the battery pack. Compared with liquid cooling, the limitations of forced air cooling are more obvious, especially when the vehicle is under the conditions of hot climates and fast-charging processes.

### 3.2. Liquid Cooling

**Figure 7:**
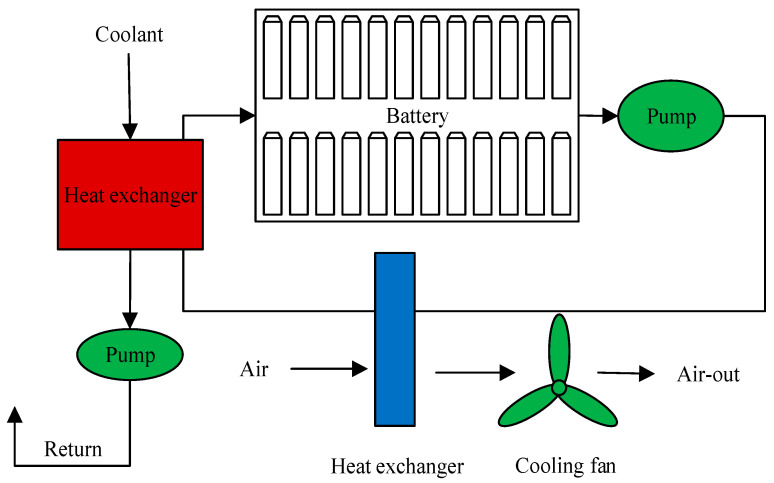
Liquid cooling system.

In liquid-cooled thermal management systems, coolant circulation (such as water, ethylene glycol aqueous solution, etc.) extracts thermal energy from the battery pack surface and transfers it to an external heat exchanger (such as a radiator), where heat is ultimately dissipated to the ambient environment. The basic working process is as follows: the coolant is pumped to the cooling channel inside the battery module or near the unit, absorbing the heat generated during battery operation; the heated coolant flows towards the heat exchanger and releases heat to the external environment through air or water cooling. After the temperature of the coolant decreases, it is circulated back into the battery module to achieve a continuous heat dissipation process, as shown in Figure 7. Compared to air-cooled cooling systems, the core of liquid cooling systems lies in their forced convection heat transfer capability, which enables liquid cooling technology to have higher heat dissipation efficiency [43]. Liquid cooling strategies are categorized into indirect and direct (immersion) cooling. Indirect cooling employs interfaces such as placing flat liquid cooling plates [44], honeycomb liquid cooling plates [45], and embedded pipes [46] positioned against the battery surface. This approach eliminates direct contact between the coolant and the battery, offering structural simplicity at the expense of an extended heat transfer path and a relatively lower cooling efficiency. In contrast, direct cooling immerses the battery in the dielectric coolant, significantly enlarging the contact area for highly efficient and uniform heat dissipation. This method, however, imposes stringent insulation requirements, necessitating the use of non-conductive fluids [47]. Considering both safety and reliability, indirect cooling is currently the mainstream method for liquid cooling, while direct cooling has not yet been widely applied in EVs.

In indirect cooling systems, the optimal liquid cooling configuration is determined by the geometry of the battery. For example, cylindrical batteries typically use embedded or honeycomb cooling pipes, while prismatic batteries typically use flat cooling interfaces. The inherent thermal resistance of the battery coolant interface caused by physical separation results in poor cooling efficiency. This limitation has prompted in-depth research into three aspects: namely, the selection of the coolant [48,49], the design of the cold plate flow channels [50], and the optimization of the system structure [51].

In terms of coolant selection, Karimi et al. [52] used water and silicone oil as a coolant to dissipate heat from the battery, and the results showed that water had better heat dissipation performance. Adding ethylene glycol solution to water can lower the freezing point and expand its usage scenarios [53]. Water–glycol systems are common in practice because they provide good cooling with manageable cost, though they present electrical safety concerns if leakage occurs. The thermal conductivity of nanofluids has drawn considerable attention in recent years. Research shows that dispersing metallic nanoparticles into fluids like water is an effective method for achieving this enhancement [54,55]. In a comparative study by Liao et al. [56] on battery-cooling performance using water and different nanofluids, copper-based nanofluids demonstrated the most effective cooling capability. The investigation revealed that increasing the volume fraction and flow rate of nanofluids resulted in a reduced maximum temperature and temperature difference in the battery. It was also found that while lowering the nanofluid temperature could decrease the maximum battery pack temperature by 10 K, this approach adversely affected thermal uniformity within the pack [57,58]. Although nanofluids can improve thermal conductivity, they suffer from particle sedimentation, increased viscosity, long-term instability, corrosion issues, and higher cost.

**Figure 8:**
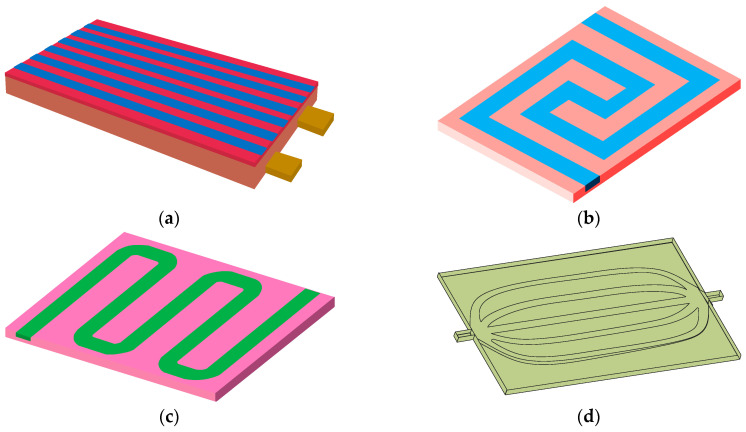
Typical flow channel design in liquid cold plate. (**a**) Parallel channel; (**b**) serpentine channel; (**c**) U-shaped serpentine channel; (**d**) streamline channel.

In terms of cold plate flow channel design, Huo et al. [59] developed a multi-channel parallel liquid cooling plate and investigated the influence of several key parameters (including channel quantity, flow direction, coolant flow rate, and ambient temperature) on both the temperature rise and thermal distribution characteristics of the battery system. The results showed that as the number of channels and flow rate increased, the maximum temperature of the battery decreased, while the flow direction had a relatively small effect on temperature. Jarrett et al. [53] proposed a serpentine channel cooling plate and performed a numerical optimization of channel width and spatial configuration. Research results showed that the optimization design of the lowest average temperature and lowest pressure drop was basically the same, that is, using the widest possible channel. However, the temperature uniformity optimization design requires the use of wider outlets and narrower inlets, which can balance the effects of the coolant flow rate, heat transfer area, and temperature gradient, thereby balancing heat transfer in all areas of the plate. On this basis, Deng et al. [60] established a U-shaped serpentine channel cold plate structure and analyzed the influence of channel quantity and arrangement on cooling performance. Simulation results showed that a five-channel arrangement in the length flow direction had the best cooling effect, and compared with a two-channel arrangement in the width flow direction, the highest temperature could be reduced by 26 °C. For cold plate flow channels, reducing the flow resistance of the coolant is an important design goal. Huang et al. [61] introduced the concept of streamline into the design of internal microchannel cooling plates, and the results showed that when using streamline design, the flow resistance was significantly reduced, improving heat transfer efficiency and temperature uniformity. The four typical liquid cooled plate flow channel designs mentioned above are shown in Figure 8. Monika et al. [50] evaluated six distinct liquid cooling plate designs of equal channel volume, which were based on typical channel structures and included linear, serpentine, U-shaped, pumpkin-shaped, spiral, and hexagonal channels. Numerical analysis found that although serpentine and hexagonal channels have a higher pressure drop, they have a good cooling performance, while pumpkin-shaped channels help reduce the pressure drop.

In terms of optimizing the structure of the cooling system, scholars have conducted research on factors such as the position and quantity of liquid cooling plates. Darcovich et al. [62] evaluated the thermal performance of cylindrical batteries by installing cold plates on both their side and bottom surfaces. Due to the larger contact area between the side cooling and the battery, the maximum temperature is lower, and the temperature uniformity is better. Similarly, Zhao et al. [63] implemented side-cooling channels in battery packs and demonstrated their capability to maintain cell temperatures within the optimal range, even under 5C charge–discharge cycles. Increasing the quantity of liquid cooling plates and rationally positioning them presents an effective approach to enhance thermal uniformity in battery packs. Qian et al. [64] found that a three-plate system optimally improved temperature distribution, lowering the peak temperature and maximum temperature difference by 13.3% and 43.3%, respectively, given a proper coolant flow rate. This inverse relationship between plate count and operating temperature was corroborated and extended by Deng et al. [51], whose systematic study of six arrangements confirmed that the pack’s maximum temperature declined as the number of plates increased. At the same time, it was found that uniformly placing cooling plates near the middle battery was more conducive to reducing the maximum temperature under the same number of cooling plates. Furthermore, topology optimization (TO) has emerged as a promising technique for enhancing the heat dissipation performance and minimizing the flow losses of liquid-cooled plates by refining the flow channel geometry. Consequently, this method has been widely adopted in cold plate research, with studies consistently affirming its efficacy in boosting cooling efficiency [65,66]. However, most related studies are based on numerical simulations. In order to explore the feasibility of TO cold plates, more experimental verification is needed in the future.

In summary, the trend of the liquid cooling is clear: complex channels provide stronger cooling, but at the expense of a higher pressure drop and pump power, while simpler channels are easier to manage but cannot meet the needs of a fast-charging or high-power operation. Nowadays, most EVs in the market use liquid cooling to dissipate the heat of the battery pack because they can achieve a reliable balance between performance and practicability.

### 3.3. Heat Pipe

**Figure 9:**
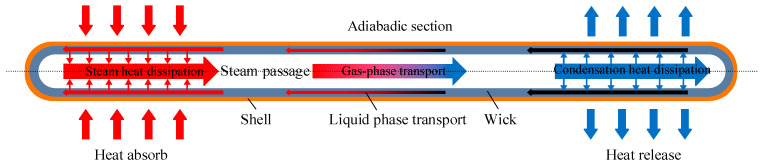
Schematic diagram of heat pipe structure.

The heat pipe (HP) is a highly efficient heat transfer device that has been widely used in battery cooling systems due to its high thermal conductivity, light weight, and high reliability. Recently, it has attracted increasing attention in the thermal management of electric vehicle batteries. The working principle of HP is based on the liquid evaporation and condensation cycle, as shown in Figure 9. Heat is transferred through phase change, and liquid reflux is achieved through capillary structure or gravity to achieve efficient heat conduction. The specific process includes four stages: (1) The evaporation stage: the evaporator section of the HP interfaces with the heat source. Upon heat generation, the working fluid within the HP absorbs thermal energy and undergoes phase change to vapor. During this evaporation process, the fluid absorbs latent heat, transforming into high-temperature, low-density vapor. (2) The steam transmission stage: the generated steam, due to pressure or density differences, rapidly moves along the tube wall with extremely low flow resistance inside the HP to the condensation section of the heat pipe. (3) The condensation stage: the condenser section interfaces with a heat sink or cooling medium. Upon reaching this section, the vapor releases its latent heat and undergoes phase transition back to liquid state, transferring thermal energy to the external environment. (4) The liquid return stage: the condensate is driven back to the evaporator section through capillary action or gravitational force within the heat pipe’s internal structure, thereby completing the cycle and preparing for subsequent evaporation–condensation processes. Heat pipes represent an innovative thermal management solution, offering unique advantages including passive operation, physical separation of heat sources and sinks, bidirectional thermal transfer capability, and switchable functionality—making them highly promising for next-generation battery cooling applications [67].

In battery cooling systems, common forms of HPs mainly include flat heat pipes, annular heat pipes, tubular heat pipes, and array micro heat pipes, etc. Scholars have conducted extensive research on them. Gan et al. [68] designed a cylindrical battery pack thermal management system based on heat pipes, which connected the battery to the heat pipe using a wavy aluminum sleeve to increase the contact area between the battery and the heat pipe. The effects of coolant flow rate, heat pipe condenser section length, and aluminum sleeve size on the temperature performance of the battery were studied through numerical analysis. Research results showed that both extension of the condensation section and increased aluminum sleeve height effectively reduced battery peak temperatures while improving temperature distribution uniformity. Liu et al. [69] developed a segmented thermal resistance model for heat pipes based on the thermal circuit methodology. Their study demonstrated that the segmented model achieved superior accuracy compared to non-segmented approaches and effectively captured the dynamic thermal behavior of heat pipe systems. This modeling approach provides a foundation for precise design, management, and control of heat pipe-based thermal management systems. Xie et al. [70] also established an embedded heat pipe model based on thermal resistance and verified the accuracy of the model through experiments. The influence of different numbers of heat pipes, fins, and heat transfer coefficients in the condensation section of heat pipes on cooling performance was studied. The results showed that increasing the number of heat pipes can reduce battery temperature and temperature difference. However, when the number of heat pipes exceeds a certain threshold, the changes in battery temperature and the temperature difference are not significant. Increasing the heat transfer coefficient and fins can also effectively reduce the battery temperature. Compared with flat heat pipes, the embedded heat pipe system has significantly better heat dissipation performance. In addition, Ye et al. [71] used a micro heat pipe array to analyze the heat dissipation of high-power lithium batteries under charge and discharge cycles. Experimental and simulation results verified the effectiveness of this structure in reducing the battery temperature rise rate and the temperature difference.

Related studies agree that heat pipes can improve temperature uniformity and reduce weight, but the effect does not scale with more pipes. Furthermore, integrating heat pipes into full battery packs is not simple. It will take time to adopt this cooling method in vehicles.

## 4. Hybrid BTMS

**Figure 10:**
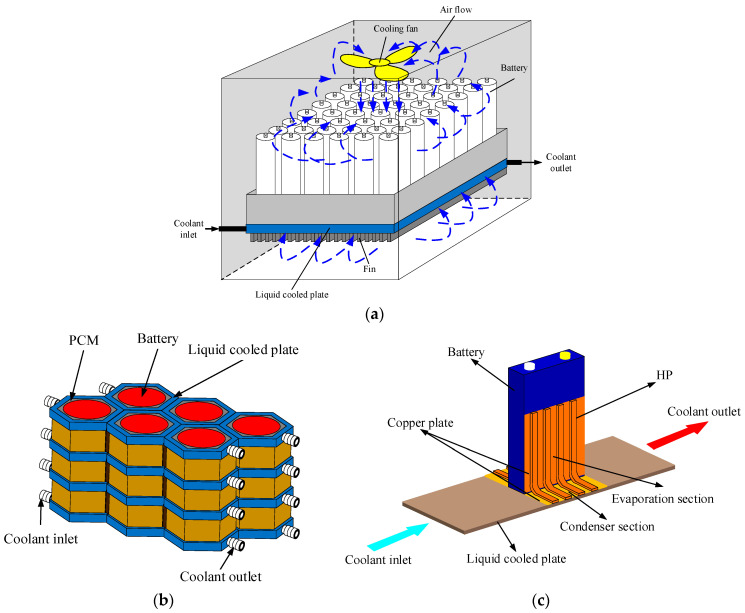
Several common hybrid cooling systems. (**a**) Liquid cooling/air cooling; (**b**) liquid cooling/PCM; (**c**) liquid cooling/heat pipe.

With the increasing demand of the market for a range of EVs and fast charging of high-power battery packs, the requirements for thermal management systems of power batteries are also becoming higher and higher. Numerous studies have shown that a single cooling method has certain limitations [43] and it is difficult to achieve satisfactory results. Therefore, hybrid BTMS combining two or more cooling methods have emerged. Due to the superior heat dissipation performance of liquid cooling systems, hybrid heat dissipation methods based on liquid cooling stand out among many hybrid heat dissipation systems, mainly including liquid cooling/air cooling, liquid cooling/PCM, and liquid cooling/heat pipe, as shown in Figure 10.

Hybrid BTMS combining air and liquid cooling require no supplementary ducting, which effectively boosts heat dissipation performance while maintaining minimal structural complexity. Wang et al. [72] proposed a hybrid cooling method, combining forced air cooling and liquid cooling plates. Studies have demonstrated the superiority of a hybrid cooling structure that positions fans below the cooling plate, considering both thermal performance and cost-effectiveness. The efficacy of combining air cooling with indirect liquid cooling to augment battery heat dissipation is corroborated by the work of Li et al. [73]. Simulation analysis revealed that although the maximum battery temperature could be maintained below 45 °C under extreme operating conditions involving elevated temperatures and high charge–discharge rates, the maximum internal temperature gradient exceeded the 5 °C safety threshold. This thermal inhomogeneity highlights limitations in this thermal management system. Zhao et al. [74] combined direct liquid cooling and forced air cooling by designing a jacket outside the battery and filling the coolant between the battery casing and the jacket.

Hybrid BTMS that integrate liquid cooling with PCM synergistically combine the merits of both: the high convective heat transfer coefficient of liquid and the high latent heat capacity and passive operation of PCM. This integration effectively reduces system energy consumption while achieving a superior cooling performance, rendering it a highly promising heat dissipating approach. Zadeh et al. [75] designed five cooling methods for a battery pack consisting of 12 lithium batteries: natural convection cooling, forced convection cooling, fin/natural convection cooling, PCM cooling, and liquid cooling/PCM hybrid cooling. Their findings indicated that the hybrid liquid/ PCM cooling system was indispensable for fulfilling the battery lifespan requirement, which mandated that operational temperatures be maintained below 45 °C. Zheng et al. [76] developed a hybrid heat dissipation system that uses PCM to fill the cooling ducts and battery gaps. Through simulation, it was found that if the heat dissipation system is mainly liquid cooled, it is difficult to utilize latent heat with PCM. They propose substituting PCM with high-thermal-conductivity materials to maintain effective system performance without relying on phase change. Conversely, to fully utilize the latent heat of PCM, Kong et al. [77] devised a hybrid cooling system. This design employs PCM as the primary cooling medium, while liquid cooling is integrated to regenerate PCM by recovering their stored latent heat, with experiments validating the system’s effectiveness and practicality.

Conventional heat pipes, relying solely on natural convection at the condensation section, demonstrate limited capability to meet battery cooling requirements under harsh operating conditions [78]. This fundamental constraint has driven the development of hybrid BTMS, integrating heat pipes with liquid cooling. Computational modeling by Mei et al. [79] confirmed that flat heat pipe-integrated liquid cooling systems substantially improve temperature uniformity across battery surfaces. Experimental validation by Yuan et al. [80] further demonstrated that a liquid-cooled/heat pipe hybrid system, with the condensation section contacting cooling plates, successfully maintained maximum battery temperature at 34.1 °C, with merely a 1 °C temperature differential under 2C discharge conditions at 30 °C ambient temperature, outperforming any single cooling method for a continuous cycling operation. In addition to the plate-contact configurations, researchers also explored the direct immersion method of the condensation section in direct contact with the coolant. Liang et al. [81] designed such a system with heat pipes embedded in liquid cooling channels and established that intermittent cooling strategies not only satisfy thermal requirements but also reduce energy consumption through optimized operational scheduling, presenting a practical approach for efficient thermal management.

**Figure 11:**
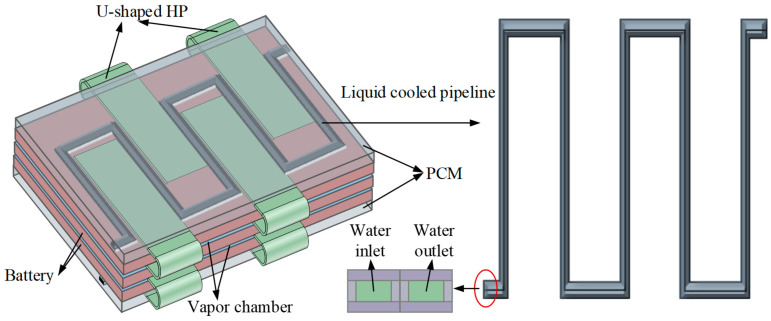
Liquid cooling/PCM/heat pipe hybrid cooling system.

To address the thermal management challenges of batteries under extreme operating conditions, researchers have developed integrated cooling systems combining liquid cooling, PCM, and heat pipes, as illustrated in Figure 11. Zhang et al. [82] developed and optimized an integrated thermal management system incorporating PCM, liquid-cooled plates, and heat pipes. Their analysis revealed that several parameters critically influence the battery’s thermal performance, including PCM thermal conductivity, PCM layer thickness, heat pipe length, and coolant inlet velocity. In addition, Li et al. [83] proposed an innovative battery thermal management system, combining thermoelectric generation (TEG) with forced convection (F-C). Research results showed that the coupled TEG-F-C system exhibited excellent temperature regulation capability under high-rate discharge conditions. This system can not only decrease the temperature of the battery module promptly but also reduce energy consumption.

It is worth noting that although hybrid BTMS consistently reduce temperature and improve uniformity, they also increase the weight, cost, and design complexity. Given these issues, hybrid BTMS are currently mainly in the theoretical research stage and have not yet been applied in mass-produced EVs.

In summary, extensive research has been conducted by scholars on battery pack thermal dissipation properties, and they have made great progress in the design of BTMS. A more intuitive comprehensive comparison of the above technologies is listed in Table 1.

## 5. Current Challenges

With the improvement of battery energy density, the evolution of fast-charging capabilities and emergence of next-generation battery systems, BTMS are facing more and more challenges. In this section, the main challenges faced by the BTMS of EVs are discussed.

### 5.1. High-Energy Density Battery and Fast Charging

The application of high-energy density batteries in electric vehicles has become a trend. However, the rapid discharge characteristics of high-energy density batteries in milliseconds pose a significant challenge to the thermal management of power batteries. Relative to conventional battery systems, high-energy density cells exhibit more localized and accelerated heat generation during operational cycles, and the heat generation rate may be several times higher than that of traditional batteries. Inadequate thermal dissipation may induce an exponential temperature rise within the battery, significantly elevating thermal runaway risks while concurrently degrading battery performance, operational lifespan, and safety. The main challenges of high-energy density batteries include: (1) A high heat generation rate: high-energy density batteries exhibit substantially greater heat generation rates compared to conventional batteries, primarily due to accelerated internal chemical reactions driven by rapid energy release over short durations [84]. (2) Uneven temperature distribution: non-uniform thermal distribution within battery packs creates localized hotspots, accelerating performance degradation and elevating safety risks [85]. (3) Risk of thermal runaway: high temperature may lead to chain chemical reactions inside the battery, resulting in electrolyte decomposition, electrode material degradation, and even a fire or explosion [86].

To address these challenges, the thermal management system must possess efficient heat dissipation capabilities and precise temperature uniformity control. However, how to achieve efficient heat dissipation in the limited space and weight is still a key problem in the development of high-power density battery technology. The hybrid liquid-air cooling system offers, in theory, adequate cooling capacity for high-power density batteries. A configuration incorporating fans directly beneath the cooling plate is particularly advantageous, as its structural simplicity eliminates the need for dedicated air ducts, achieving an optimal compromise between performance and cost.

**Figure 12:**
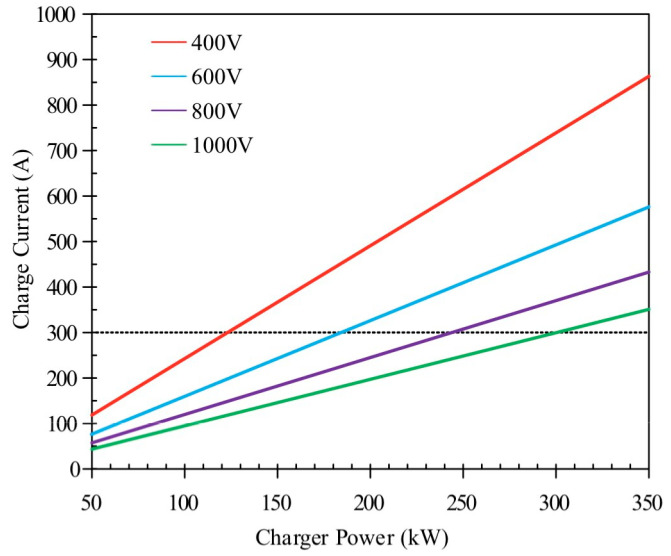
Relationship between charging current and charging power of different battery pack voltages. Reprinted from Ref. [87].

In order to support the fast-charging performance of the batteries, a lot of energy has been invested in single-module battery pack configuration. It should be noted, however, that a battery pack constitutes a complex system comprising not only individual cells but also electrical interconnects, BTMS, and other essential components. While individual cells exhibit excellent fast-charge capability and power density, system integration at pack level often compromises these advantages while substantially increasing costs [87]. In the process of high-power charging, the current will also increase, which will induce rapid thermal accumulation within the cell, leading to the decline in battery performance, capacity attenuation, and even the risk of thermal runaway. Consequently, the current is constrained to 400 A. For typical EV power batteries, this threshold is only attainable at charging voltages exceeding 1000 V (Figure 12.), necessitating enhanced thermal management capabilities to maintain safe operating temperatures during high-rate charging and power-intensive discharge cycles [14]. As mentioned earlier, in direct cooling systems, the coolant contacts the battery directly, substantially increasing the heat transfer area. This results in high heat transfer efficiency and uniform cooling: a combination that is essential for managing the intense and rapid heat generation during fast charging. Consequently, this method can effectively suppress thermal runaway and minimize temperature gradients, directly addressing the primary thermal challenges associated with high-power charging protocols.

Moreover, the BTMS does not operate in isolation, but interacts critically with the vehicle’s HVAC (Heating, Ventilation and Air Conditioning) system, often competing for refrigerant and electrical power, particularly under extreme conditions, necessitating a synergistic design to concurrently manage battery temperature and cabin comfort without overloading the electrical system. Long-term durability and reliability of the BTMS are also paramount, as its performance must be consistently maintained over the vehicle’s lifespan to ensure ongoing battery safety and longevity.

### 5.2. New-Type Battery Technology

The emergence of new battery technology brings new challenges to the thermal management system. For example, solid-state batteries and lithium–sulfur batteries, recognized for their high-energy density and enhanced safety characteristics, have emerged as pivotal development pathways for next-generation electric vehicle energy storage systems.

Solid-state batteries are considered to be an important direction for the next generation of battery technology, because of their advantages of high-energy density, high safety, and long cycle life. However, solid-state batteries exhibit limited thermal conductivity, which brings new challenges to the design of its thermal management system. Compared with the traditional liquid electrolyte battery, the electrolyte in a solid-state battery usually has low thermal conductivity, resulting in low heat conduction efficiency inside the battery, which easily causes local overheating [88]. Therefore, the design of solid-state batteries requires careful consideration of both thermal interface materials and cooling system architecture to ensure optimal performance. The challenges are reflected in the following aspects: (1) poor thermal conductivity: the thermal conductivity of solid electrolyte is usually low, resulting in uneven heat distribution inside the battery, increasing the risk of thermal runaway [89]; (2) poor interface stability: the stability of the interface between the electrode and electrolyte of the solid-state battery is poor, which is easy to form a high impedance thermal interface, further hindering the heat conduction [90]; (3) high thermal interface impedance: resulting from insufficient physical contact between the solid electrolyte and electrodes, the thermal interface impedance is high, which imposes more stringent requirements on the design of thermal management systems [91]. To meet these challenges, researchers are exploring a variety of solutions. For example, by developing solid electrolyte materials with high thermal conductivity (such as a sulfide-based or oxide-based electrolyte), the heat transfer capability within batteries is improvable [92]. In addition, optimizing battery structures, such as using multilayer composite electrodes or introducing thermal interface materials, can effectively reduce the thermal interface impedance and improve the heat dissipation efficiency [93]. The thermal management efficiency of solid-state batteries can be substantially improved through the synergistic application of heat pipe and PCM cooling technologies. Through optimized integration of solid-state batteries with thermal management systems, enhanced heat dissipation, prolonged cycle life, and improved safety can be simultaneously achieved [94,95,96].

Lithium–sulfur batteries have become a strong competitor for the next generation battery technology due to their high theoretical energy density (about 2600 WH/kg) and low cost. However, lithium–sulfur batteries still face many challenges at the application level, especially in terms of thermal management, including the following aspects: (1) polysulfide dissolution and migration: in the process of charge and discharge, the positive pole of lithium–sulfur batteries will generate polysulfides. These polysulfides migrate to the negative electrode and react with the lithium metal, resulting in the loss of active substances and capacity attenuation [97]. Furthermore, polysulfide dissolution and migration contribute to internal heat accumulation [98,99]; (2) poor thermal stability: lithium–sulfur batteries exhibit inherent thermal instability, especially when discharging at high power, a considerable amount of thermal energy is generated within the battery. Delayed heat dissipation may trigger rapid temperature escalation, potentially initiating thermal runaway [100]; (3) temperature sensitivity: lithium–sulfur battery systems exhibit strong temperature-dependent performance characteristics. At a low temperature, the ionic conductivity of the battery decreased significantly, leading to performance degradation; at high temperatures, the dissolution and migration rates of polysulfides accelerate, further aggravating the risk of capacity decay and thermal runaway [101]. To meet these challenges, researchers have proposed three solutions to these three aspects: (1) polysulfide inhibition technology: using functional diaphragms or electrolyte additives to inhibit the dissolution and migration of polysulfides, thereby reducing heat accumulation [102]; (2) efficient heat dissipation design: by using heat-management technologies, such as liquid cooling or PCMs, transfer heat to the external environment to avoid over-heating [103]; (3) temperature monitoring and control: by combining with a real-time temperature monitoring system and dynamic temperature control strategy, adjust the charging and discharging rate in time to prevent thermal runaway [104].

In summary, for new power batteries such as solid-state batteries and lithium sulfur batteries, their thermal management schemes are not limited to the conventional heat dissipation design but also need to consider the physical and chemical properties of battery materials. For solid-state batteries, due to the absence of liquid electrolyte, thermal management focuses more on interface optimization and lightweight heat dissipation structure. For lithium sulfur batteries, additional suppression of polysulfide diffusion is required, and their thermal management needs to balance chemical stability and heat dissipation efficiency. In the future, the thermal management of solid-state batteries and lithium–sulfur batteries may develop towards a composite solution of combining liquid cooling with PCM cooling or combining heat pipes with an intelligent algorithm.

## 6. Summary and Outlook

With the continuous progress of battery technology, the future battery thermal management technology will develop in a more efficient and intelligent direction. Thermal regulation system design for battery packs will become increasingly important for improving battery performance and prolonging service life.

### 6.1. Technical Summary

At present, thermal management systems for EV batteries mainly focus on three ways: passive BTMS, active BTMS, and hybrid BTMS.

The passive BTMS regulate the temperature of the battery through natural physical processes. The main advantages of these technologies are low energy consumption, low cost and simplicity, but they may face some limitations in the application of high-energy density and fast charging.

The active BTMS uses external energy sources (such as fans, pumps, and coolants) to actively regulate the battery temperature. Compared with the passive BTMS, the active BTMS can provide stronger cooling capacity, which is especially suitable for a high-power battery pack and fast-charging environment. The common active BTMS technologies include forced air cooling, liquid cooling, and heat pipe cooling. Forced air cooling configurations remain prevalent in low-power applications, owing to their structural simplicity and cost-effectiveness. Liquid cooling systems, especially in high-power battery pack, have become the mainstream technology. Liquid cooling strategies are categorized into indirect and direct (immersion) cooling. Considering both safety and reliability, indirect cooling is currently the mainstream method for liquid cooling, while direct cooling has not yet been widely applied in EVs. The trend of the liquid cooling is clear: complex channels provide stronger cooling at the expense of the higher pressure drop and pump power, while simpler channels are easier to manage but cannot meet the needs of fast charging or high-power operation. Heat pipes can improve temperature uniformity and reduce weight, but the effect does not scale with more pipes. Furthermore, integrating heat pipes into full battery packs is not simple and we still need some time to adopt this cooling method in vehicles.

Hybrid BTMS, combining two or more cooling methods, can consistently reduce battery temperature and improve the uniformity of battery temperature. Due to the superior heat dissipation performance of liquid cooling systems, hybrid heat dissipation methods based on liquid cooling stand out among many hybrid BTMS, mainly including liquid cooling/air cooling, liquid cooling/PCM, and liquid cooling/heat pipe. Although hybrid BTMS can consistently reduce temperature and improve uniformity, they also increase the weight, cost, and design complexity. Given these issues, hybrid BTMS are currently mainly in the theoretical research stage and have not yet been applied in mass-produced EVs.

### 6.2. Future Directions

Future thermal management technology for batteries will revolve around the four core areas, which are efficient heat dissipation, intelligent regulation, lightweight structure, and material innovation, combined with the development of new technologies, such as solid-state batteries, to achieve a balance between safety, endurance, and cost. Specific research can be conducted on the following four aspects.

The first direction is to achieve global integration of vehicle energy management and low-carbon energy conservation. The thermal management system will shift from “single component optimization” to “global collaborative management”, integrating temperature control of the batteries, motors, electronic controls, and passenger cabins, and achieving optimal energy consumption through intelligent algorithms. For example, apply heat pump technology to intelligent thermal management of EVs to improve energy utilization efficiency.

The second direction is to achieve intelligent prediction and control of the battery temperature. By analyzing user habits (such as commuting routes, charging time) and environmental data (temperature, sunshine) through the intelligent connected vehicle data, pre-adjustment of the batteries’ temperature can be achieved. For example, automatically preheating the battery at low temperatures can save charging costs, and enhancing heat dissipation power in advance at high temperatures can reduce energy consumption and extend battery life.

The third direction is the embedded cooling structure design. In the future, cooling channels will be directly integrated into battery modules (such as double-sided cooling cells, filling gaps with phase change materials) or use bottom integrated liquid cooling plates to improve the heat dissipation efficiency while reducing the volume and weight of the battery packs.

The fourth direction is the innovation and application of new materials. The new materials include PCM, high thermal conductivity interface materials, and high-temperature-resistant electrolytes. PCM use paraffin or other PCM to absorb heat and stabilize the temperature, alleviating an instantaneous temperature rise. High thermal conductivity interface materials can optimize the thermal resistance between the battery and the heat-dissipation structure and then improve conduction efficiency. High-temperature-resistant electrolytes, such as oxide/sulfide solid electrolytes, can suppress high-temperature side reactions. Therefore, researching how to safely and efficiently apply these materials to the cooling system of power batteries is an important development direction.

## Figures and Tables

**Table 1:** Summary of BTMS.

References	Technology Type	Advantages	Disadvantages
Ling et al. [23] Ping et al. [24] Weng et al. [25] Jiang et al. [26] Nasehi et al. [27]	PCM cooling	Low cost Providing temperature uniformity High efficiency Simple structure	Leakage problem Low thermal conductivity Heavyweight Flammability
Yang et al. [30] Du et al. [31] Peng et al. [32] Kang et al. [33] Sun et al. [34] Chen et al. [35] Lu et al. [36] Liu et al. [37]	Air cooling	Low cost Simple structure High reliability Easy maintenance	Low efficiency Low cooling capacity Noise (when using fan)
Karimi et al. [52] Al Shdaifat et al. [55] Liao et al. [56] Panchal et al. [57] Malik et al. [58] Huo et al. [59] Deng et al. [60]	Liquid cooling	Better efficiency Higher cooling capacity Flexibility in design Controllability Reduced noise (Compared to air cooling)	Complex structure Leakage problem Need a large space Heavyweight
Gan et al. [68] Liu et al. [69] Xie et al. [70] Ye et al. [71]	Heat pipe	Efficient thermal conductivity High reliability Flexible structure	High cost High design complexity Risk of fluid leakage
Wang et al. [72] Li et al. [73] Zhao et al. [74] Zadeh et al. [75] Zheng et al. [76] Kong et al. [77] Mei et al. [79] Yuan et al. [80] Zhang et al. [82]	Hybrid system	Adaptable to complex working conditions High efficiency	High cost Difficulty in maintenance Compatibility issues

## Data Availability

Not applicable.
